# Machine Learning-Based Models for Prediction of Toxicity Outcomes in Radiotherapy

**DOI:** 10.3389/fonc.2020.00790

**Published:** 2020-06-05

**Authors:** Lars J. Isaksson, Matteo Pepa, Mattia Zaffaroni, Giulia Marvaso, Daniela Alterio, Stefania Volpe, Giulia Corrao, Matteo Augugliaro, Anna Starzyńska, Maria C. Leonardi, Roberto Orecchia, Barbara A. Jereczek-Fossa

**Affiliations:** ^1^Division of Radiotherapy, IEO European Institute of Oncology IRCCS, Milan, Italy; ^2^Department of Oncology and Hemato-Oncology, University of Milan, Milan, Italy; ^3^Department of Oral Surgery, Medical University of Gdańsk, Gdańsk, Poland; ^4^Scientific Directorate, IEO European Institute of Oncology IRCCS, Milan, Italy

**Keywords:** radiotherapy, toxicity, predictive models, machine-learning, radiomics

## Abstract

In order to limit radiotherapy (RT)-related side effects, effective toxicity prediction and assessment schemes are essential. In recent years, the growing interest toward artificial intelligence and machine learning (ML) within the science community has led to the implementation of innovative tools in RT. Several researchers have demonstrated the high performance of ML-based models in predicting toxicity, but the application of these approaches in clinics is still lagging, partly due to their low interpretability. Therefore, an overview of contemporary research is needed in order to familiarize practitioners with common methods and strategies. Here, we present a review of ML-based models for predicting and classifying RT-induced complications from both a methodological and a clinical standpoint, focusing on the type of features considered, the ML methods used, and the main results achieved. Our work overviews published research in multiple cancer sites, including brain, breast, esophagus, gynecological, head and neck, liver, lung, and prostate cancers. The aim is to define the current state of the art and main achievements within the field for both researchers and clinicians.

## Introduction

It is estimated that as many as half of the cancer patients in the world are eligible for radiotherapy (RT), either with curative or palliative intent (1). Ultimate generation linear accelerators and modern techniques, such as intensity-modulated RT (IMRT), stereotactic body RT (SBRT), and proton therapy (PT), offer high conformity and submillimetric levels of precision. However, normal tissues close to the target region, defined as organs at risk (OARs), can also be affected, leading to RT-induced toxicity. Short-term or acute toxicity occurs during treatment or within 3 months after its completion, and generally, full recovery occurs within weeks to months. Conversely, late effects, such as fibrosis or RT-induced oncogenesis, are generally considered as irreversible and progressive over time. It follows that, when planning any RT treatment, its potential benefits have to be weighed against the possibilities of damage to healthy organs and tissues, with the final aim of maximizing curative response while minimizing the probability of normal tissue complications. On the other hand, when RT is delivered with curative intent, target coverage should not be jeopardized in favor of sparing OARs (2). However, different RT-induced side effects vary in their clinical significance, so an accurate estimate of risks is mandatory, especially when alternatives such as surgery or chemotherapy are available. The physiopathology of toxicity is not only related to the radiation dose but also depends on genetic factors and tumor microenvironment. Therefore, identifying the main factors that predispose for a specific type of toxicity can help to improve treatment planning and inform patients and clinicians about expected treatment tolerance.

Radiosensitivity is generally studied with the so-called normal tissue complication probability (NTCP) models, which can be classified into mechanistic (or analytical) and data-driven [or (semi)empirical] (3). The former category is based on a simplified characterization of the interaction between radiation and biological tissues and seeks to explain the underlying mechanisms with explicit algorithms. The most common analytical models are the Lyman–Kutcher–Burman models, which are often included into treatment planning systems to allow for a biological optimization of the delivered dose among competing treatment strategies (4). These algorithms are based on handcrafted rules with intricate exceptions that often fail to predict the actual complications induced by RT. On the other hand, data-driven approaches are based on the assumption that the interaction between radiation and normal tissue is complex and cannot be properly represented deterministically. Therefore, such approaches aim to identify the model that best fits the input data (also termed features or independent variables) and output data (also termed response or dependent variables). Predictors of toxicity can be roughly classified into “dosimetric,” which directly concerns the delivery of radiation (e.g., dose-volume histogram (DVH) points), “clinical,” which includes patient- and disease-related variables (e.g., gender and tumor histology), and “image-based” or “radiomic,” which can be extracted from various medical images (e.g., the mean, variance, and skewness of image intensity histograms). In general, these approaches can be further distinguished into well-known traditional statistical techniques, such as regression-based techniques, and approaches based on artificial intelligence (AI) and machine learning (ML) (5).

### ML-Based Models of Toxicity

**Figure 1:**
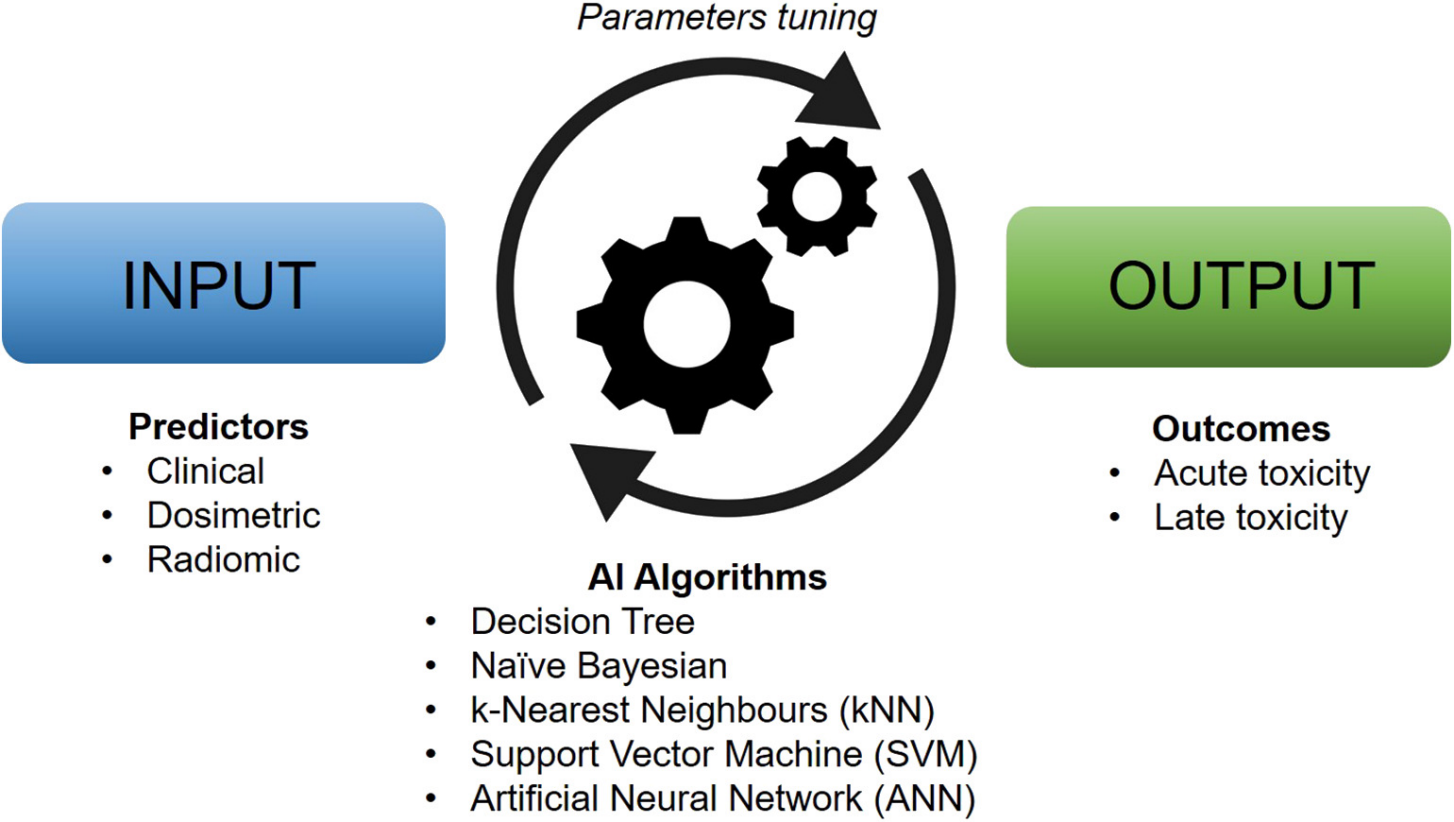
Typical workflow of artificial intelligence-based models for clinical toxicity prediction. Machine learning algorithms work by tuning their characteristic parameters by modeling the relationship between input and output data in an automatic manner.

The theoretical framework for artificially intelligent ML models was laid down already in the 1950s (6), but it was not until recently that advances in technology have allowed for the integration of these tools into the experimental and clinical practice of health sciences. AI, in its broadest sense, denotes an artificial system able to perform a certain task to some success. ML, typically considered a subset of AI, generally refers to some set of algorithms that can “learn” to perform a specific task without explicit implementation of the solution (although the terms AI and ML are often used interchangeably). For instance, ML algorithms are able to produce predictions on new and unseen data after being trained on a finite learning data set and are especially useful for tasks that involve a large amount of data or variables (Figure 1). With the plethora of possible variables that can lead to toxicity, ML approaches are particularly well suited to model the relationship between treatment-induced side effects and related covariates. An ML model that is able to predict an outcome from a set of inputs, after tuning the best set of parameters on a number of training cases, is referred to as a classifier. Some common classifiers are naïve Bayes, logistic regression (LR), k-nearest neighbors (kNN), random forests (RF), support vector machine (SVM), and artificial neural networks (ANN).

Since the ML model will learn the parameters from the available data, it follows that the characteristics of the data set are absolutely crucial. If the training data set is sparse, the model typically fails to learn a representative set of parameters that can be generalized to instances outside of the data set. This problem, which generally arises when a model has been trained to encompass a particular set of data too closely, is known as overfitting or overtraining. Overfitting can occur for a variety of reasons and should always be a major concern when constructing an ML model.

Since the performance of any ML model depends on the particular problem and data set it is applied to, it is intractable to generally rank different methods. Nevertheless, an acceptable approximation of a model's performance is given by the so-called AUC (which is defined as the area under the receiver operating characteristic curve) applied to an independent validation set. The AUC value of a model ranges between 1, corresponding to perfect classification of the validation set, and 0.5, corresponding to a purely random classification. It is important to note, however, that the AUC can be severely misleading in case of flaws in the model design, such as heavily imbalanced data sets or misused validation procedures.

Successful ML models have the potential to aid clinical facilities and practitioners in minimizing side effects and increasing the likelihood of positive outcomes. Despite a good amount of research in ML methods for toxicity assessment, to the best of our knowledge, this is the first effort to summarize the current state of the field. Previous publications have focused either on specific anatomical districts (5) or exclusively on methodologies and theory (7, 8). Therefore, the aim of this review is to present an overview of current achievements in the field as well as main areas of debate and possible future directions, both from a methodological and a clinical perspective.

## Search Strategy and Selection Criteria

A comprehensive literature review was performed through the use of a search string (see Supplementary Materials S1) built by an experienced medical librarian with input from the study investigators. Different combinations of database-specific terms were used, supplemented by keywords in order to cover all the areas related to RT toxicity, ML, and toxicity prediction. The literature review was conducted using the PubMed/Medline databases in order to identify publications to be synthetized into an exhaustive overview of the state of the art of ML application for the prediction of RT-induced toxicity. The search resulted in 864 hits. Reference lists of selected articles were hand searched for further potential relevant papers and also using the Snowballing technique (9). Studies with no focus on cancer, radiation therapy, toxicity, or any kind of ML (in its broadest sense) were excluded, together with articles dealing with pediatric patients. All publications in languages other than English were also excluded. In the end, 53 studies were included in this narrative review. The search was conducted in March 2020 (see Supplementary Materials S2).

## Overview of Contemporary Research

Many studies were found that employ ML-based models to predict RT-related side effects. Most of them concern head and neck (H&N) (13 studies), lung (15 studies), and prostate (16 studies) cancers, while a minor portion focused on brain (1 study), breast (3 studies), esophagus (1 study), gynecology (3 studies), and liver (1 study) cancers (Table 1). The presented literature is divided into different sections according to the anatomical district. Focus was put into presenting both methodological and clinical aspects of the papers.

**Table 1:** Summary of reviewed literature.

**Cancer type**	**References**	**No. of pts**	**Type of RT**	**Type of predicted toxicity**	**Features type**	**Classifier**	Results^*^
Breast	(10)	90	RT	Dermatitis	R	RF	Acc = 0.87 (test)
	(11)	2277		Moist desquamation, dermatitis, chest pain, fatigue	D, C	LR, RF, gradient boosting	0.56–0.85
	(12)	827	RT	Telangiectasia	D, C	LASSO	
Esophagus	(13)	101	IMRT or 3D-CRT	Pneumonitis	D, C	LR	Acc = 0.63
Gyneco	(14)	42	EBRT+BRT	Rectal toxicity	D	SVM	0.82–0.91
	(15)	42	EBRT+BRT	Rectal toxicity	D	CNN (transfer learning)	1.29
	(16)	35	BRT	Fistula formation	D, C	SVM	1.30
H&N	(17)	437	RT (397) PT (40)	Toxicity (grade ≥3)	C	LR, RF, XGBoost	0.63–0.65
	(18)	2121	RT	Unplanned hospitalizations, Feeding tube placement, Weight loss	D, C	LR, gradient boosting, RF	0.64–0.76
	(19)	153	RT	Xerostomia	D, R, C	6 ML algotithms	Best SVM and extra-trees 0.74–0.89
	(20)	86	RT	Trismus	D	IBDM	Identification of a cluster of voxel related with toxicity
	(21)	427	RT	Xerostomia	D, C	LR, LASSO, RF	Best LR (0.70)
	(22)	173	RT	Acute dysphagia	D, C	SVM, RF	0.82
	(23)	297	IMRT	Xerostomia (grade ≥2)	D, C	LR	Model updating is beneficial
	(24)	134	IMRT and PT	Esophagitis	R, D	LASSO	0.75
	(25)	47	3D-CRT	Sensorineural hearing loss	R, C	Decision stump, Hoeffding	76.08% accurarcy 75.9% precision
	(26)	37	IMRT	Parotid shrinkge Xerostomia	D, C	Fuzzy logic Naïve Bayes	Acc = 0.79–0.86
	(27)	249	IMRT	Xerostomia, sticky saliva	R, D	Multivariate LR	0.77
	(28)	351	IMRT	Mucositis	D, C	LR, SVM, RF	0.71 (RF)
	(29)	1 (H&N) 1 (Prostate)	IMRT	Xerostomia (H&N), Rectal bleeding (prostate)	D	Decision tree, SVM	0.42% MAE (H&N) 97% acc (prostate)
Liver	(30)	125	SBRT	Hepatobiliary toxicity	D, C	CNN (transfer learning)	1.25
Lung	(31)	110	SBRT	LC, DFS, OS, and fibrosis	R	Cox regression	
	(32)	203	IMRT or PT	Pneumonitis	C	RF	1.06
	(33)	192	IMRT and 3D-CRT	Radiation pneumonitis	R, D, C	LASSO	0.68
	(34)	197	SBRT	Chest wall syndrome	D, C	Descision tree RF	n/a
	(4)	3496 (lung+brain +H&N)	RT	Classifiers comparison	D, C	Decision tree, RF, ANN, SVM, elastic net, logit-boost	Best: elastic net LR and RF
	(35)	14	SBRT	Lung injuries	R, D	LR	0.64–0.78
	(36)	201	SBRT	Pneumonitis	D, C	Decision trees, RF, RUSBoost	
	(37)	115	RT	Esophagitis	D, C	LASSO	0.78
	(38)	54	3D-CRT	Pneumonitis	D, C	Bayesian network LR Single variable	0.66–0.83
	(39)	748	RT	Esophagitis	D, C	LR	0.83
	(40)	219	3D-CRT	Pneumonitis	D, C	SVM	1.16
	(41)	55 (H&N) 219+166 (Lung)	3D-CRT	Xerostomia, Pneumonitis (166) Esophagitis (216)	D, C	LR, SVM, ANN	Best: modified SVM
	(42)	219	RT	Radiation pneumonitis	D, C	Decision tree, ANN, SVM, self-organizing maps	0.79
	(43)	234	RT	Radiation pneumonitis	D, C	Decision tree	0.72
	(44)	166	EBRT	Esophagitis xerostomia	D	LR	
	(45)	142	3D-CRT	Pneumonitis	D	ANN	0.61–0.85
Prostate	(46)	64	IMRT (52 pts), 3D-CRT (12 pts)	Urinary toxicity Gastro-intestinal toxicity	R, D, C	LR	0.65–0.77
	(47)	33	IMRT	Cystitis	R	LR	0.62–0.75
	(48)	33	IMRT	Rectal wall changes	R	LR	0.46–0.81
	(49)	351	RT	Rectal bleeding Fecal incontinence Urinary incontinence Nocturia	R, D, C	LR	0.58–0.73
	(50)	598	RT	Late fecal incontinence	D, C	ANN	0.78
	(51)	593	RT	Rectal bleeding	D, C	ICA	0.83, 0.80, 0.78
	(52)	324	BRT+-EBRT	GU toxicity symptoms	D, C, G	RF	0.7
	(53)	118	EBRT, BRT	GI toxicities	D	LR	Identification of spatial constraint for toxicity reduction
	(54)	368	RT	Rectal bleeding, Erectile dysfunction	C, G	RF, LR	0.71 (rectal bleeding) 0.68 (erectile dysfunction)
	(55)	79	IMRT	Rectal toxicity (grade ≥2)	D, C	LR	1.28
	(56)	754	EBRT	Dysuria, hematuria, incontinence, frequency	D, C	LR, Elastic-net, SVM, RF, ANN, MARS	Best: LR, MARS AUC = 0.65
	(57)	99	EBRT	Rectal bleeding	D	LDA, SVM, k-means, kNN, PCA, CP-DMA	Best: CP-DMA
	(58)	261	3D-CRT	Rectal toxicity, rectal bleeding	D, C	RF NTCP, NTCP	0.76, 0.66
	(59)	718	RT	Rectal bleeding		LR, ANN	0.655, 0.704
	(60)	321	RT	Acute bladder and rectal toxicity	D, C	ANN, SVM	0.7
	(61)	119	RT	Rectal bleeding Nocturia	D	ANN	Sensitivity and specificity >55%

*3D-CRT, 3D conformal RT; Acc, accuracy; ANN, artificial neural network; AUC, area under the curve; BRT, brachytherapy; CNN, convolutional neural network; CP-DMA, canonical polyadic decomposition–deterministic multi-way analysis; DFS, disease free-survival; EBRT, external beam RT; GI, gastrointestinal; GU, genitourinary; H&N, head and neck; IBDM, image-based data mining; ICA, indipendent component analysis; IMRT, intensity-modulated RT; kNN, k-nearest neighbors; LASSO, Least Absolute Selection and Shrinkage Operator; LC, local control; LDA, linear discriminant analysis; LR, logistic regression; MAE, mean absolute error; MARS, multivariate adaptive regression splines; ML, machine learning; NTCP, normal tissue complication probability; n/a, not applicable; OS, overall survival; PCA, principal component analysis; pt, patient; PT, proton therapy; RF, random forest; RT, radiotherapy; RUSBoost, random under-sampling Boost; SBRT, stereotactic body RT; SVM, support vector machine. Features were classified as clinical (C), dosimetric (D), genomic (G), or radiomic (R)*.

* *If not specified, AUC values are reported*.

### Brain

A single study on ML-based toxicity modeling was found related to brain cancer (4). In the study, the authors conducted a comprehensive comparison of the performance of different ML classifiers on multiple data sets including patients with brain, lung, and H&N primaries. Their models included decision trees, RF, neural network, SVM, elastic net LR, and Logit-Boost classifiers and were tested on 12 distinct data sets for a total of 3496 patients. Both dosimetric and blood marker data from meningioma as well as (non)-small-cell lung cancer (NSCLC) and H&N cancer patients were considered. No single classifier was found to be ideal across all data sets, but RF and net LR performed comparably (best in six and four data sets, respectively). Based on these results, the authors also investigated methods of preselecting a classifier, concluding that empirical selection of the classifier is advantageous, leading to an average AUC increase of 0.02.

### Breast

Current available literature includes only one abstract (11) and two full papers (10, 12). In the study by Saednia et al., they proposed an innovative approach based on the detection of body-surface temperature increase induced by radiation dermatitis. Thermal images of the irradiated breast were taken from a pool of 90 patients at four consecutive time points: pre-RT and after 5, 10, and 15 fractions, respectively (with a total dose of 42.50 Gy in 16 fractions). Skin toxicity was assessed at the end of RT with the Common Terminology Criteria for Adverse Events (CTCAE) guidelines. On the independent testing data set, the RF classifier showed a good accuracy (87%) at the fifth fraction in predicting the skin toxicity at the end of RT.

The authors in the study by Reddy et al. trained three different classifiers, namely, RF, gradient boosted decision tree, and LR, on a large population of 2277 patients to predict the occurrence of common radiation toxicities, such as moist desquamation, radiation dermatitis, breast/chest wall pain, and fatigue. Validation performances reached AUC values of 0.85, 0.82, 0.77, and 0.56 for the respective endpoints. According to the authors, it was the first demonstration of the ability to accurately predict acute RT toxicities in a prospective validation data set.

Finally, Mbah et al. set out to highlight the main failure causes for models predicting RT-induced toxicity. Data from two different German cohorts were used for a total of 827 breast cancer patients who received RT. The Least Absolute Selection and Shrinkage Operator (LASSO) LR model was used to predict telangiectasia within each individual data set separately. Each model was also externally tested on the other data set. To their surprise, they found that one predictive variable (hypertension) had a positive coefficient on one data set, and a negative coefficient on the other. Some variables were also exclusive to a specific model, thus suggesting that overcoming overfitting does not completely solve the problem of generalization.

### Esophagus

An ML-based model for toxicity prediction in esophagus cancer patients was published by Hart et al. (13). In their work, the authors investigated the relationship between clinical symptoms of radiation pneumonitis and the pulmonary metabolic activity on post-treatment [^18^F]-fluorodeoxyglucose positron emission tomography (FDG PET). Their study included a cohort of 101 patients who underwent restaging FDG PET/computed tomography (CT) imaging between 3 and 12 weeks after completing thoracic RT for esophageal cancer. Several LR models were built with different combinations of treatment and dosimetric variables, obtaining a peak accuracy of 0.63 with *p* ≤ 0.032 when combining pulmonary metabolic radiation response with the mean lung dose, thus indicating a significant relationship between pulmonary metabolic radiation response and radiation pneumonitis.

### Gynecological Cancers

The three studies in this section analyze toxicity outcomes prediction following brachytherapy alone or in combination with external beam RT (EBRT) in gynecological cancers. All the models were trained with limited data sets, ranging between 35 and 42 patients, and with SVM or convolutional neural network (CNN) classifiers.

Tian et al. (16) developed a model for fistula formation prediction with an SVM classifier. Thirty-one different features were used as predictor variables from a relatively small sample of 35 patients treated with interstitial brachytherapy. Their model reached a high accuracy of 0.901, but the authors rightfully point out the strong limitation deriving from the usage of the small data set.

One study by Chen et al. (14) investigated the relationship between rectal toxicity (CTCAE grade ≥2) and dosimetric features. In detail, the feature calculation was performed on both the 3D rectum surface and the 2D deformed accumulated rectal surface dose map. The models, for which they used SVM classifiers, achieved AUC values of 0.82 and 0.91 for different feature selection procedures (and 42 patients). The authors also demonstrated that the ML model outperformed classification based on the conventional Groupe Européen de Curiethérapie-European SocieTy for Radiotherapy & Oncology (GEC-ESTRO) dosimetric parameters Dose to 0.1, 1 and 2 cm^3^, which achieved an AUC of 0.71.

Zhen et al. (15) tested the feasibility of a CNN for rectum toxicity prediction through a transfer learning approach. The network itself, originally developed by the visual geometry group at the University of Oxford, had been pretrained on the ImageNet data set. The fine-tuning step was then performed on unfolded rectum surface dose maps (RSDM). By using the gradient-weighted class activation maps, the authors were also able to identify the existence of discriminative regions on the RSDM. Their results demonstrate than the CNN can outperform conventional dosimetric parameters with top AUC values of 0.89 as compared to a meager 0.58 for the one-dimensional dose-volume (DV) parameters (or 0.7 for 2D RSDM features). The authors also presented comparisons between the transfer learned network and a network trained from scratch.

### Head and Neck

The size of the training data sets in published works on H&N cancers ranges from 37 to 2121 patients. Predicted toxicity outcomes included late xerostomia, acute mucositis, parotid shrinkage, unplanned hospitalization, and weight loss. Applied classifiers included LR, RF, gradient boosting, and one based on fuzzy logic. In addition, one study (4) made a comparison of the performance of different classifiers on different data sets (please refer to the Brain section for further details).

The two most recent articles (17, 18) both applied three different classifiers (RF, gradient boosting, and LR models) to predict unplanned hospitalizations, feeding tube placement, and significant weight loss (Reddy) and grade ≥3 toxicity (Wojcieszynski). Reddy et al. considered a large data set of 2,121 patients, comparing over 700 treatment-related and clinical variables, and achieved AUC values of up to 0.640, 0.755, and 0.751 for RF, gradient boosting, and LR, respectively. Wojcieszynski et al. achieved a moderate success in predicting grade ≥3 toxicity for 437 patients after 90 and 180 days (*c*-statistic 0.65 and 0.63, respectively) using 47 different patient covariates. Among them, planning target volume (PTV) integral dose, body mass index (BMI), integral dose to regions outside the PTV, and age were most statistically impactful ones.

By retrospectively comparing updating strategies, Nakatsugawa et al. (23) demonstrated the importance of continuous model revising. On their data set, they concluded that the best strategy was to update the model yearly, keeping only the two most recent years of data. The method they used was LR classifying grade ≥2 late xerostomia with clinical and dosimetric variables from 297 patients.

The aim of the study by Beasley et al. (20) was to identify specific CT image regions with a dose–toxicity association to identify radiation-induced trismus in H&N patients treated with RT. To achieve this objective, an image-based data mining (IBDM) framework was applied to a cohort of 86 patients. The IBDM approach allowed for the identification of a cluster of voxels associated with trismus; this cluster was internally validated using a DVH-based approach and externally on a cohort of 35 patients. As stated by the authors, this study represents the first clinical application of IBDM with a continuous outcome variable.

Jiang et al. (21) utilized a data set of 427 H&N cancer patients treated with RT to predict xerostomia. Ridge LR, LASSO LR, and RF classifiers were trained with planned radiation dose data and non-dosimetric features to investigate the influence of dose patterns on xerostomia. Among the three different ML methods explored, ridge LR showed the best predictive power with an AUC of 0.70, although the difference in performance was not statistically significant. The study highlighted how radio-morphology combined with ML methods can indicate the patterns of dose which are most influential on xerostomia, potentially improving radiation treatment planning.

Dean et al. (22) developed a model to predict severe acute dysphagia in H&N cancer patients treated with RT. Penalized LR (PLR), SVM, and RF models were trained using dosimetric and clinical data and then internally and externally validated on 173 and 90 patients, respectively. Results showed that PLR model performances were comparable with the more complex models with an AUC of 0.82 and that dose to the pharyngeal mucosa was an important predictor of dysphagia.

In another study, Gabryś et al. (19) investigated whether xerostomia risk assessment can be amended by ML with dosimetric, radiomic, and demographic features, rather than only using a NTCP model. The authors compared predictive performance of seven classification algorithms, six feature selection methods, and 10 data cleaning/class balancing techniques using the Friedman test and the Nemenyi *post-hoc* analysis. A cohort of 153 H&N cancer patients was used to predict xerostomia at different time stages. Their multivariate models achieved AUC values ranging from 0.74 to 0.88, with SVM and “extra-trees” having the top performances. The authors also pointed out that LR was preferred for univariate feature selection, and that data cleaning/class balancing had no advantage. Their NTCP models, on the other hand, failed to predict xerostomia (AUC <0.6).

The study of Abdollahi et al. (48) aimed to predict sensorineural hearing loss in radiochemotherapy-treated H&N cancer patients. From a cohort of 47 patients, 490 image features of 94 cochlea were derived from CT images. To perform feature selection, classification, and prediction, 10 different ML approaches were tested. The predictive power (AUC, accuracy, and precision) of the ML algorithms was over 0.70 in all cases; the best was obtained by Decision Stump and Hoeffding modeling with 76.08% and 75.9% accuracy and precision, respectively. In conclusion, CT radiomic analysis, both with and without clinical and dosimetric variables, could help with chemoradiation-induced hearing loss.

On a small data set of 37 patients treated with IMRT, Pota et al. (26) applied a fuzzy logic-based classifier in order to predict the occurrence of parotid shrinkage and 12-month xerostomia. To do this, they used clinical features, dosimetric parameters, CT-based radiomic features, and combinations thereof as predictor variables. They achieved high respective accuracies of up to 0.86 (parotid shrinkage) and 0.79 (xerostomia). Their developed model is easily interpretable and have comparable performance to a naïve Bayes classifier.

The goal of the study by Van Dijk et al. (27) was to build a predictive model for xerostomia and sticky saliva in H&N cancer patients using CT image biomarkers (IBMs). The planning CT scans of 249 H&N cancer patients were collected to extract IBMs in order to create multivariable LR models, which were then internally validated by bootstrapping. In total, 26 features correlated with xerostomia and 24 correlated with sticky saliva were selected. The results showed how the addition of IBMs of the parotid and submandibular glands to dosimetric data improved the mean AUC from 0.74 to 0.77. The authors found that the IBM “short run emphasis” was the most important for xerostomia prediction, and “maximum CT intensity” was the most important for sticky saliva prediction. These features represented heterogeneity and density within the salivary glands, respectively.

Dean et al. (28) compared LR, SVM, and RF classifiers in a framework to predict severe acute mucositis on a cohort of 351 patients. Their variables included dose-volume (DV) parameters, spatial dose metrics, and clinical data. Although model performances were comparable, the best performance was obtained with the RF classifier, with an AUC value of 0.71. The authors also confirmed that reducing the volumes of oral cavity receiving intermediate/high doses may reduce mucositis incidence.

Zhang et al. (29) developed decision tree and SVM models for a single H&N patient. The model was supposed to predict saliva flow rate with DV constraints and tailored plan properties as input variables. The mean absolute error of predicting saliva flow rate was 0.42%. Their results suggest that “ML tools can be used to guide planners to select DV constraint settings corresponding to all involved OARs in a knowledge-driven manner.”

El Naqa et al. (41) investigated several types of linear and non-linear kernels^1^ to generate interaction terms and approximate the treatment-response function in order to capture the potential complexity of heterogeneous variable interactions more accurately. This study investigated xerostomia on a data set with 55 H&N cancer patients as well as two data sets with prostate cancer (PCa) patients. By first analyzing patient distributions with principal component analysis (PCA), they concluded that SVM outperformed both LR and an ANN.

### Liver

Ibragimov et al. (30) employed a pre-trained CNN model on 3D dose maps in order to predict liver toxicity after SBRT. They also included non-dosimetric patient variables as additional inputs to the network. By using the saliency maps of the network, they were able to identify anatomical regions that are critical to spare during SBRT. On their data set of 125 patients, their model managed to predict hepatobiliary toxicity with an AUC of 0.85. In addition, their deep learning model also predicted almost two times fewer false-positive toxicity cases compared to DVH-based predictions. The authors also observed that irradiation of the proximal portal vein was associated with two times higher toxicity risks than irradiation of the left portal vein.

### Lung

For lung cancers, the size of the data sets ranged between 54 and 235 patients. The majority of the studies dealt with radiation-induced pneumonitis, whereas some studies dealt with esophagitis, xerostomia, sticky saliva, and chest pain. Lung cancer RT may cause chest pain due to rib fracture, radiation-induced neuropathy of the intercostal nerves or nerve branches, chest wall edema, or chest wall fibrosis. However, the only study we found that specifically investigated chest pain is the one by (34). The authors utilized decision tree and RF methods to identify robust features predictive of chest wall pain in a cohort of 197 patients. Both univariate and multivariate analyses confirmed the role of rib dose to 1 cc, chest wall dose to 30 cc, and rib dose max (*D*_max_) as relevant variables. Based on these findings, efforts should be directed at lowering the rib dose to 1 cc <4000 cGy, chest wall dose to 30 cc <900 cGy, and rib *D*_max_ <5100 cGy in order to mitigate chest wall syndrome.

Das et al. performed two studies (42, 43) for pneumonitis prediction in a data set of 219 lung cancer patients treated with RT. In both studies, the final model derived from a fusion of two or more single models. In the study dated 2007, starting from a data set of 234 lung cancer patients treated with RT, they trained a model for lung radiation-induced grade 2+ pneumonitis. The model consisted of a parametric dose-based Lyman NTCP model in conjunction with weighted non-parametric decision trees. The combined models' predictive power resulted in an AUC of 0.72—an improvement compared to the 0.62 AUC of the Lyman NTCP alone. In particular, the information about non-dose variables provided by the decision trees could add interpretability and aid in dissemination. In the study dated 2008, the authors constructed a consensus model by fusing four different non-linear multivariate models: decision trees, neural networks, SVMs, and self-organizing maps. Consensus was achieved by simply averaging the predictions for each patient from all four individual models (in an ensemble-wise manner, i.e., with several predictions for each individual model). This achieved an average AUC value of 0.79 with lower variance than the individual component models.

Esophagitis is another common side effect in lung cancer RT, but only two studies researched this topic (41, 44). In the former, the authors explored model building and variable selection methods for multivariate dose-response assessment, considering a data set of 166 NSCLC patients. Using a LR classifier, the authors concluded that performance can be improved by mixing clinical and DV factors as input parameters. In the second paper, they investigated several types of linear and non-linear kernels to approximate the treatment-response function and capture the potential complexity of heterogeneous variable interactions. This was done with a data set of 219 lung cancer patients. In the same article, the authors also investigated pneumonitis on a data set of 166 patients and xerostomia on a data set of 55 patients. After applying PCA to analyze variable distributions, they concluded that SVM outperformed both LR and an ANN.

Niedzielski et al. (24) explored a novel method for using CT imaging biomarkers to quantify patients' radiosensitivity and subsequently predict esophagitis risk. Patients with high response to radiation, despite lower radiation dose, were labeled as radiosensitive. This information was extracted through K-means clustering (an automatic clustering algorithm) with three nodes. The authors concluded that inclusion of the radiosensitive variable improved LASSO LR model performance (mean AUC, 0.75) compared to models without this information (mean AUC, 0.69). Their predictive model was built with a cohort of 134 NSCLC patients treated with IMRT (85 pts) or passive-scatter PT (49 pts).

Valdes et al. (36) developed a patient-specific “big data” clinical decision tool in order to predict radiation-induced pneumonitis in stage I NSCLC patients who received SBRT. In the study, the performance of three different algorithms [Decision Trees, RF, random under-sampling (RUS) Boost] was evaluated on a cohort of 201 lung cancer patients. The feature selection highlighted that the most important features for pneumonitis prediction were the diffusion capacity of the lung for carbon monoxide and the dose to the heart, trachea, and bronchus. The authors also stated that at least 800 patients are needed to keep the error below 10% for pneumonitis prediction.

Huang et al. performed two studies for prediction of esophagitis. In the first one (39), a model for the assessment of severe acute esophagitis for NSCLC patients treated with RT was constructed. Correlation analysis and LR models with clinical and dosimetric variables were tested on three different Washington University data sets including a total of 748 patients. Their most successful bivariate model (using the variables mean esophagus dose and concurrent chemotherapy) achieved an AUC of 0.83. In the second one (37), they tested the previously published model to predict the risk of severe acute esophagitis on a new independent data set of 115 NSCLC patients. The model used a logistic function with the same two predictor variables: mean esophageal dose and concurrent chemotherapy. When comparing the model with a new model built solely on the independent data set, the authors concluded that the former was almost as predictive as the latter (although the same variables were selected), being AUC = 0.78.

Most of the published studies concern radiation-induced pneumonitis as the target variable, as it represents one of the principal dose-limiting toxicities associated with thoracic RT (40). Of these studies, Lee et al. (38) developed a Bayesian network approach in a cohort of 54 NSCLC patients treated with 3D conformal RT (3D-CRT). For inference, they included DV, clinical, and blood biomarker data. They also compared the Bayesian network ensemble approach, which managed to achieve an AUC of 0.83, with a LR classifier (AUC = 0.77), and univariate predictors (AUC ≤ 0.69). Valdes et al. (36) considered a larger data set of 201 stage I NSCLC patients to construct different models with decision trees, RF, and RUSBoost, concluding that RUSBoost had the best performance. They found that the three most important predictive features were the dose to 15 cc of the heart, dose to 4 cc of the trachea or bronchus, and race. However, rather than developing a model for clinical use, the article focused on the power of using learning curves and comparisons of testing and training error to guide the discovery process.

Su et al. (45) investigated an approach to build an ANN, comparing three different validation methods. The ANN was built as a fully connected three-layered feed forward network, and achieved peak AUC values of 0.85. As input to the network, they used DV data from a data set of 142 patients treated with 3D-CRT. Chen et al. (40) tested an SVM model in a data set of 219 patients and compared two models: one including only dose variables (AUC = 0.71), while the other used dose as well as non-dose variables (AUC = 0.76). They concluded that it is indeed beneficial to include non-dose factors in prediction. The two most predictive variables in their model were generalized equivalent uniform doses close to the mean lung dose, and chemotherapy prior to RT. Luna et al. (32) used a RF approach in a cohort of 203 patients treated with stage II–III locally advanced NSCLC. They evaluated 32 clinical features at both univariate and multivariate analysis and confirmed the importance of lung volume receiving 20% of dose (V20), lung mean, and pack-year as predictors of radiation pneumonitis. They also identified esophagus max as a new possible indicator.

Beside dosimetric- and clinical-based predictors, image-based variable models have also been employed to predict RT-related toxicity outcomes in lung cancer patients. Bousabarah et al. (31) used CT-based radiomic features to predict radiation-induced lung injuries. The study analyzed 110 patients with primary stage I/IIa NSCLC treated with stereotactic body RT for predicting various outcomes, including local lung injury up to fibrosis. Interestingly, for this classification task, only first-order features from gray-level histogram were found to be predictive. Overall, the work suggested that radiomic analysis of planning CT images may help to predict local lung injury up to fibrosis, together with disease-free survival and overall survival in lung cancer patients treated with SBRT. The derived features can be regarded as imaging biomarkers that could support the clinical decision process to the benefit of the patients and oncologist.

Moran et al. (35) investigated the potential of CT-based radiomic features to characterize post-SBRT lung injury. They also investigated the relationship between changes of radiomic feature values and accumulated dose by constructing dose–response curves. The ability to assess lung injury was tested by using a logistic regression classifier, which achieved AUC values in the 0.64–0.75 range using only gray level co-occurrence matrix (GLCM) features. Their results showed that eight out of nine features demonstrated a significant dose–response relationship, suggesting a potential objective measurement of post-SBRT lung injury.

Krafft et al. (33) developed a predictive model for radiation pneumonitis using CT-extracted radiomic features in combination with clinical and dosimetric parameters from a cohort of 192 NSCLC patients. Of the 192 patients, 80% (152) were treated with IMRT while the remainder with 3D-CRT. A LASSO logistic regression classifier was built, resulting in an average AUC of 0.68, showing an increased performance compared to models not including image features (AUC = 0.51).

### Prostate

The most common toxicity outcomes in PCa RT are erectile dysfunction (ED), gastrointestinal (GI) disorders, rectal toxicity, and genitourinary (GU) side effects. To predict these unwanted outcomes, the reviewed studies trained several different ML classifiers including SVM, ANN, RF, and multivariate adaptive regression splines (MARS) with data sets of sizes between 79 and 754. Lee et al. (52) also took a gene ontology analysis into account to identify biological processes related to radiation-induced toxicity and predicted late GU toxicity symptoms in a cohort of 324 PCa patients. In this study, the only clinically valid model, which achieved an AUC of 0.7, was for predicting weak stream with RFs. The genetic analysis they conducted highlighted neurogenesis and ion transport as key biological processes related to urinary tract functions.

The study by Carrara et al. (50) was designed to predict late fecal incontinence in PCa patients treated with RT, using ANN classification methods. A population of 598 PCa patients was tested, recording information about comorbidities, previous abdominal surgeries, drug treatments, and dose distribution. In order to identify the best-performing ANNs, the authors varied the number of inputs and neurons and simulated a great amount of ANN configurations. Finally, the best ANN model was selected, showing an 80.8% sensitivity and 63.7% specificity in late fecal incontinence prediction, with an AUC of 0.78.

Fargeas et al. (51) applied an independent component analysis (ICA) model to predict RB in a cohort of 593 PCa patients treated with RT. Two subspaces from the rectal DVHs (with and without RB) were identified and integrated with dosimetric and clinical parameters in a Cox proportional hazards model for RB prediction. The model was tested for 3, 5, and 8 years RB prediction, with AUCs of 0.68, 0.66, and 0.64, respectively. Interestingly, when ICA parameters were included the model, performances increased with new AUCs of 0.83, 0.80, and 0.78.

In their paper, Oh et al. (54) developed a novel classification algorithm that they call pre-conditioned random forest regression (PRFR). The algorithm was tailored for genome-wide association studies based on single-nucleotide polymorphisms (SNPs). On their cohort of 368 PCa patients treated with RT, the aim was to construct a predictive model of two post-RT clinical endpoints: rectal bleeding and ED. After generating a SNP importance score, they included the top 50% most relevant SNPs in their model. This procedure achieved AUC values of 0.71 and 0.68 for rectal bleeding and ED, respectively, outperforming traditional classification algorithms such as RF and logistic regression. The authors also concluded that the model performance could be further improved by incorporating clinical variables.

Moulton et al. (53) investigated the relationship between spatial dose distribution and GI toxicities including rectal bleeding, stool frequency, diarrhea, and tenesmus. Their study contained data from 118 patients treated with a combined EBRT/high-dose-rate brachytherapy treatment. By building models with logistic regression and the Wilcoxon signed rank test, they were able to investigate the association between dose surface map-related features and toxicities. Their findings indicated that spatial constraints on doses to certain sections of the rectum may be important for reducing toxicities and optimizing the dose.

Both Liu and Li (55) and Pella et al. (60) modeled acute grade rectal toxicity for PCa patients using dosimetry and patient clinical characteristics after treatments with IMRT and 3D-CRT, respectively. The model by Liu achieved a significatively better AUC (0.88) when clinical and dosimetric variables were combined, as compared to a model considering only dosimetric features (0.67). In particular, the use of statin drugs and prostate-specific antigen (PSA) level prior to IMRT was found to be strongly related to the toxicity outcome. Pella et al. instead compared an ANN model with an SVM model trained with dosimetric and clinical data from 321 patients treated with conformal RT. The results obtained showed comparable performances of up to 0.7 AUC for the two compared models.

Yahya et al. (56) conducted a classifier comparison for different urinary symptoms on a cohort of 754 PCa patients. With dose-surface data, comorbidities, and medication intake as input parameters, they analyzed the clinical endpoints dysuria, hematuria, incontinence, and frequency. The following classifiers were compared: LR, elastic-net, SVM, RF, neural network, and MARS. They pointed out that the predictive power is endpoint-dependent and modest at best (AUC = 0.65). Best performance was found for LR and MARS, although elastic-net and RF gave comparable results.

Fargeas et al. (57) developed a novel approach that they call CP-DMA to predict patients presenting rectal bleeding. The name CP-DMA comes from *canonical polyadic decomposition*, an alternative name for tensor rank decomposition, and *deterministic multi-way analysis*. The model uses tensor rank decomposition of the fourth-order tensors created by 3D dose distributions concatenated for different patients (in the fourth dimension) in order to find two separate vector subspaces (one subspace for each outcome, with or without rectal bleeding). Patients are then classified according to their distance to the respective subspaces. Results were compared to linear discriminant analysis, SVM, K-means, kNN, a PCA-based unsupervised algorithm, unsupervised multidimensional classification, and an NTCP model. Their model achieved an AUC of 0.85, outperforming the alternative methods.

Ospina et al. (58) compared the performances of a classical NTCP model with a RF NTCP model for late rectal toxicity prediction on a cohort of 261 patients with PCa treated with 3D-CRT. Both clinical and dosimetric features were collected to train three RF models in order to predict three different 5-year rectal toxicity endpoints: grade 2 overall rectal toxicity and grade 1 and 2 rectal bleeding. Performance of the model ranged between 0.66 and 0.76 depending on the toxicity endpoint. Authors highlighted that the most suitable parameters to be considered in rectal toxicity prediction include dose to the rectum, age, and anticoagulant treatment of the patients.

Zhang et al. (29) developed decision tree and SVM models for one PCa patient (as well as a H&N cancer case), predicting rectal bleeding (RB) with DV constraints and tailored plan properties as input variables. The RB prediction had an average accuracy of 97.04%, indicating that the selection of DV constraint setting can be guided with ML methods.

The study by Tomatis et al. (59) aimed to compare the performances in predicting late RB in a cohort of 718 PCa patients of an LR model and an ANN one using clinical and DVH-based parameters. Overall, the ANN model outperformed the other, with AUCs of 0.704 vs. 0.655, respectively. Authors suggested how the integration of gene expression profiles and surface dose mapping could help to improve the predictive performances of the model.

Gulliford et al. (61) were early adopters of ANN for predicting biological outcomes following PCa RT. They used the treatment plan prescription and dose distribution data in order to predict rectal bleeding and nocturia on a data set with 119 patients. Analysis was made on different discretization levels of the outcomes, and an attempt was made to “look inside” the ANN at a basic level. Their results showed sensitivities and specificities of roughly 0.55.

Several studies aiming to correlate radiomic features with toxicity outcomes are present in the literature. In the study by Mostafaei et al. (46), the potential role of CT radiomics to predict prostate RT toxicities, including acute bladder and rectal injuries, was investigated. Sixty-four PCa patients were studied. The findings highlighted the feasibility and good performance of pre-treatment CT image features as new markers to predict radiation toxicities. The results also showed that, for cystitis, the combination of radiomic features with clinical and dosimetric features could enhance the predictive performance: from AUC values of 0.71 and 0.67 for radiomic and clinical models alone, to AUC = 0.77 when the features were combined. However, for proctitis modeling, the performance was lower in the combined setup compared to the radiomics-only model (AUCs for clinical, radiomic, and clinical–radiomic models were 0.66, 0.71, and 0.65, respectively). These results suggest that integration of radiomics with clinical and dosimetric features may improve the performance of predictive models.

Abdollahi et al. (47) analyzed magnetic resonance imaging (MRI) images from a pool of 33 patients in order to predict urinary toxicity in PCa patients. Different radiomics features (S5.0SumVarnc, S2.2SumVarnc, S1.0AngScMom, S0.4SumAverg, and S5.5InvDfMom) were tested, resulting in AUC values between 0.62 and 0.75 and showing a major dependence of radiomic features on radiation dose. Overall, feature changes resulted to have a good correlation with radiation dose and radiation-induced urinary toxicity. These radiomic features can be identified as being potentially important imaging biomarkers which can also allow to assess mechanisms of radiation-induced bladder injuries.

Abdollahi et al. (25) applied radiomic feature analysis on pre/post IMRT MRI images to find imaging biomarkers for rectal toxicity prediction. Feature extraction was made on both T2-weighted and apparent diffusion coefficient (ADC) images (two different MRI scanning protocols). Pre-IMRT T2-weighted radiomic image features could predict rectal toxicity with a fairly good performance (AUC mean: 0.68), showing a better predicting power in relation to ADC image features (AUC mean: 0.58). The AUC reached 0.81 when all features were combined, suggesting that pre-treatment MRI features may be a feasible approach to predict radiation-induced early rectal toxicity.

Finally, Rossi L. et al. (49) applied DVH parameters, texture features of patients' 3D dose distributions, and non-treatment-related (NTR) predictors to develop predictive models for GI and GU toxicities. Multivariate LR models were trained using the NTR features alone as well as in combination with the other variables. RB, fecal incontinence, nocturia, and urinary incontinence were considered. For RB, fecal incontinence, and urinary incontinence, AUC values increased when adding DVH and texture features to NTR features (from 0.58, 0.63, and 0.68 to 0.73, 0.73, and 0.73, respectively). In the case of nocturia, inclusion of DVH parameters resulted in a marginal improvement (0.64 vs. 0.66). Overall, the inclusion of more features improved prediction performance for GI and GU toxicity.

## Discussion

In recent years, the growing interest toward AI in all fields of science has led to the development of innovative tools in RT (62), including several toxicity prediction models. Some of them have demonstrated high performance on very large and diverse data sets, making them potential candidates for clinical integration. Other ones have highlighted cases where ML prediction seems to fail, such as in predicting unplanned hospitalizations or fatigue. Interestingly, almost half of the 53 reviewed papers were published in the last 3 years, with the earliest publication dating back to 2004, making it a rather young area of interest with much potential for future research.

Our overview indicates that the amount of research on ML-based models for prediction of toxicity is not balanced across districts, as some of them, such as lung, prostate, and H&N have been receiving more attention than others such as brain, skin, blood, and breast. Regarding brain cancer, the lack of ML models is potentially ascribable to the scarcity of literature in general concerning radio-induced toxicity within the brain. This may be explained by the fact that acute and late complications of brain tumor patients prevalently manifest themselves as neurological disorders that are difficult to assess. On the other hand, H&N studies are common mainly because these kinds of cancers, albeit not as common as PCa or lung cancer, are very often associated with clinically relevant toxicity, with a well-documented impact on patients' quality of life. Additionally, accurate prediction of RT toxicity in H&N cancer may help physicians to identify the best treatment option whenever equally effective approaches (i.e., surgery) are available. Furthermore, integration of genetic information in the modeling approaches, despite being desirable, appears almost completely absent, being treated only in two studies (52, 54).

The large variety of variables, features, and models, as well as the lack of standardization in the development of predictive tools, accounts for the scarce comparability of the existing works. As previously pointed out, performance measures such as the AUC are not the be-all and end-all of model assessment and should be taken with a grain of salt. The AUC measure has even been criticized as an indicator of performance altogether (63) and can sometimes be misleading. For instance, out of all the selected papers, the best results (AUC > 0.85) were achieved in small- or medium-sized data sets (<150 patients). This implies that further validation of the current best-performing models on larger and/or more diverse data sets is mandatory.

Since the principal aim of ML models for toxicity prediction is clinical integration, critical efforts are required to make the relevant research understandable, transparent, and accessible to an audience with little or no specific computational background. As a matter of fact, considering the specific case of this review, the studies did not always accurately report clinical information concerning pathology, RT treatment (technique, dose, fractionation scheme), the kind of developed toxicity (late or acute), as well as methodological details (feature selection procedures and employed models). Therefore, a rigorous method for communicating characteristics and results of prediction models, which would foster the synthesis and critical appraisal of the relevant information, is of paramount importance. One of them was proposed by the Transparent Reporting of a multivariable prediction model for Individual Prognosis Or Diagnosis (TRIPOD) initiative (64), which consists of a checklist that encompasses a minimum set of details that authors should fulfill to provide essential and clear information about their work. In particular, the key points should include a summary of objectives, study design, setting, participants, sample size, predictors, outcomes, statistical analysis, results, and conclusions. This would ensure that proper assessment of usefulness, potential biases, and possible drawbacks of published research can be made.

Other open issues are the importance of data sharing among centers, the need for continuous model updates, and the need for prospective studies to support the clinical applicability of the developed models. More research and effort in these areas will alleviate the issue of clinical integration, which represents both the primary driver and the ultimate goal of these efforts.

## Conclusion

Despite the loose ends about the clinical applicability of RT-induced toxicity models, our overall findings show that ML-based solutions for toxicity prediction in RT could represent a valid tool in research settings. In order to maximize the therapeutic index of RT and to guide the clinical selection of patients, an effective toxicity prediction scheme is essential. Application of such models can be a valuable asset in many different aspects for both patients and clinicians.

## Author Contributions

LI, MP, MZ, GM, and BJ-F were responsible for conception and design of the study and wrote the first draft of the manuscript. SV was responsible for data acquisition and wrote sections of the manuscript. MA, DA, GC, AS, ML, and RO wrote sections of the manuscript. All authors contributed to manuscript revision, and read and approved the submitted version.

## Conflict of Interest

The authors declare that the research was conducted in the absence of any commercial or financial relationships that could be construed as a potential conflict of interest.

## Footnotes

^1^In this context, kernels are mathematical transformation functions that allow an implicit embedding of data in another feature space. For the purpose of this article, different kernels can be thought of as different types of SVMs.

## Abbreviations

3D-CRT: 3D conformal RT
ADC: apparent diffusion coefficient
AI: artificial intelligence
ANN: artificial neural network
AUC: area under the curve
BMI: body mass index
BRT: brachytherapy
CNN: convolutional neural networks
CP-DMA: canonical polyadic decomposition–deterministic multi-way analysis
CT: computed tomography
CTCAE: common terminology criteria for adverse events
Dmax: dose max
DV: dose-volume
DVH: dose-volume histogram
EBRT: external beam RT
ED: erectile disfunction
EORTC: European Organization for Research and Treatment of Cancer
FDG PET: [^18^F]-fluorodeoxyglucose PET
GEC-ESTRO: Groupe Européen de Curiethérapie-European SocieTy for Radiotherapy &
Oncology
GI: gastrointestinal
GLCM: gray level co-occurrence matrix
GU: genitourinary
H&N: head and neck
IBM: image biomarker
IBDM: image-based data mining
ICA: independent component analysis
IMRT: intensity-modulated RT
kNN: k-nearest neighbors
LASSO: Least Absolute Selection and Shrinkage Operator
LR: logistic regression
MARS: multivariate adaptive regression splines
ML: machine learning
MRI: magnetic resonance imaging
NSCLC: non-small-cell lung cancer
NTCP: normal tissue complication probability
NTR: non-treatment related
OAR: organ at risk
PCa: prostate cancer
PCA: principal component analysis
PET: positron emission tomography
PLR: penalized logistic regression
PRFR: pre-conditioned random forest regression
PSA: prostate-specific antigen
PT: proton therapy
PTV: planning target volume
RB: rectal bleeding
RF: random forest
RSDM: rectum surface dose maps
RT: radiotherapy
RUS: random under-sampling
SBRT: stereotactic body RT
SNP: single nucleotide polymorphism
SVM: support vector machine
TPS, treatment planning system. TRIPOD: Transparent Reporting of a multivariable prediction model for Individual Prognosis Or Diagnosis
V20: volume receiving 20% of dose.
