# Supplementary material for: Machine Learning-Based Models for Prediction of Toxicity Outcomes in Radiotherapy
Source: Front Oncol. 2020 Jun 5;10:790. doi: 10.3389/fonc.2020.00790 (PMC7289968; doi:10.3389/fonc.2020.00790)
Supplement: Supplementary file 1 [file Data_Sheet_1.docx]

**S1. Search string for the review**

**((((((("Artificial Intelligence"[Mesh] OR "Logistic Models"[Mesh]) OR ("artificial neural network"[All Fields] OR "artificial neural networks"[All Fields] OR "computer neural network"[All Fields] OR "computer neural networks"[All Fields] OR "artificial intelligence"[All Fields] OR "deep learning"[All Fields] OR "machine learning"[All Fields] OR "support vector machine"[All Fields] OR "support vector machines"[All Fields] OR (support[All Fields] AND ("genetic vectors"[MeSH Terms] OR ("genetic"[All Fields] AND "vectors"[All Fields]) OR "genetic vectors"[All Fields] OR "vector"[All Fields] OR "disease vectors"[MeSH Terms] OR ("disease"[All Fields] AND "vectors"[All Fields]) OR "disease vectors"[All Fields]) AND machinery[All Fields]) OR "random forest"[All Fields] OR "naive bayes"[All Fields] OR "bayes classification"[All Fields] OR "decision tree"[All Fields] OR "decision trees"[All Fields])) OR ("logistic regression"[All Fields] OR "logistic regressions"[All Fields] OR (logistically[All Fields] AND regressed[All Fields]) OR "logistic model"[All Fields] OR "logistic models"[All Fields] OR "predictive model"[All Fields] OR "predictive models"[All Fields])) OR "Logistic Models"[Mesh]) OR ("logit model"[All Fields] OR "logit models"[All Fields])) AND ((("Radiation Effects"[Mesh] OR "radiation effects"[Subheading]) OR "Radiation Pneumonitis"[Mesh]) OR ("radiation injury "[All Fields] OR "radiation injuries"[All Fields] OR "radiation toxicity" OR radiotoxicity OR "radiation pneumonitis"[All Fields] OR "radiation esophagitis"[All Fields] OR "radiation oesophagitis"[All Fields] OR "radiation effect"[All Fields] OR "radiation effects"[All Fields]))) AND (((("adult"[MeSH Terms] OR "adult"[All Fields]) OR ("adult"[MeSH Terms] OR "adult"[All Fields] OR "adults"[All Fields]) OR ("aged"[MeSH Terms] OR "aged"[All Fields] OR "elderly"[All Fields])) OR ("Aged"[Mesh] OR "Middle Aged"[Mesh])) OR "Middle Aged"[All Fields])) NOT (("child"[MeSH Terms] OR "child"[All Fields]) OR ("Childhood"[Journal] OR "childhood"[All Fields]) OR ("child"[MeSH Terms] OR "child"[All Fields] OR "children"[All Fields]) OR ("infant"[MeSH Terms] OR "infant"[All Fields]) OR ("infant"[MeSH Terms] OR "infant"[All Fields] OR "infants"[All Fields]) OR ("infant, newborn"[MeSH Terms] OR ("infant"[All Fields] AND "newborn"[All Fields]) OR "newborn infant"[All Fields] OR "newborn"[All Fields]) OR ("pediatrics"[MeSH Terms] OR "pediatrics"[All Fields] OR "pediatric"[All Fields]) OR ("pediatrics"[MeSH Terms] OR "pediatrics"[All Fields] OR "paediatric"[All Fields]) OR ("paediatrics"[All Fields] OR "pediatrics"[MeSH Terms] OR "pediatrics"[All Fields]) OR ("paediatrics"[All Fields] OR "pediatrics"[MeSH Terms] OR "pediatrics"[All Fields]))**
