# Supplementary material for: Machine Learning-Based Models for Prediction of Toxicity Outcomes in Radiotherapy
Source: Front Oncol. 2020 Jun 5;10:790. doi: 10.3389/fonc.2020.00790 (PMC7289968; doi:10.3389/fonc.2020.00790)
Supplement: Supplementary file 2 [file Data_Sheet_2.doc]

**PRISMA Flow Diagram**

**Screening**

**Included**

**Eligibility**

**Identification**

Records identified through librarian search
(n = 831)

Records already included identified through other sources
(n = 33)

All records
(n = 864)

Records after duplicates were removed (n = 798)
(n = 798)

Records excluded
(n = 709)

Full-text articles assessed for eligibility
(n = 95)

Full-text articles excluded, with reasons
(n = 42)

No machine learning (n = 28)

No toxicity prediction (n = 17)

Studies included in the narrative review
(n = 53)
